# Spin-lattice-dynamics analysis of magnetic properties of iron under compression

**DOI:** 10.1038/s41598-023-41499-2

**Published:** 2023-08-31

**Authors:** Gonzalo dos Santos, Robert Meyer, Diego Tramontina, Eduardo M. Bringa, Herbert M. Urbassek

**Affiliations:** 1 CONICET and Facultad de Ingeniería, Universidad de Mendoza, Mendoza, 5500 Argentina; 2 Physics Department and Research Center OPTIMAS, University Kaiserslautern-Landau, Erwin-Schrödinger-Straße, 67663 Kaiserslautern, Germany; 3 Centro de Nanotecnología Aplicada, Facultad de Ciencias, Universidad Mayor, Santiago, 8580745 Chile

**Keywords:** Physics, Condensed-matter physics, Materials science, Condensed-matter physics, Theory and computation

## Abstract

Compression of a magnetic material leads to a change in its magnetic properties. We examine this effect using spin-lattice dynamics for the special case of bcc-Fe, using both single- and poly-crystalline Fe and a bicontinuous nanofoam structure. We find that during the elastic phase of compression, the magnetization increases due to a higher population of the nearest-neighbor shell of atoms and the resulting higher exchange interaction of neighboring spins. In contrast, in the plastic phase of compression, the magnetization sinks, as defects are created, increasing the disorder and typically decreasing the average atom coordination number. The effects are more pronounced in single crystals than in polycrystals, since the presence of defects in the form of grain boundaries counteracts the increase in magnetization during the elastic phase of compression. Also, the effects are more pronounced at temperatures close to the Curie temperature than at room temperature. In nanofoams, the effect of compression is minor since compression proceeds more by void reduction and filament bending—with negligible effect on magnetization—than by strain within the ligaments. These findings will prove useful for tailoring magnetization under strain by introducing plasticity.

## Introduction

The ferromagnetism of bulk bcc-Fe has since long been investigated using ab-initio techniques^1^. These also allow to understand the local effect of isolated point defects—such as vacancies and interstitials^2–4^—and also of high-symmetry defect structures such as low-index surfaces^5^ and grain boundaries^6–9^. However, the effect of extended defects—in particular dislocations—and also of structures extending over more than a few nm, is beyond the computational reach of ab-initio techniques. In the last decade the method of spin-lattice dynamics (SLD) has obtained microscopic understanding by coupling atomistic molecular dynamics simulation of the lattice with a classical description of the dynamics of the spin system^10–13^.

Compression of metals induces plasticity which is based on extended defects such as dislocations and twins. The interaction of compression and magnetism requires the coupled investigation of magnetic and mechanical properties of Fe. Such a coupling was recognized long time ago^14^. Nowadays, it is well known that magnetization and plasticity can influence each other^15–17^ and that an understanding of the coupling can help in engineering desired magnetic and mechanical properties. Recently Li et al.^18^ used density-functional-theory calculations to describe the decrease in magnetization in an Fe lattice under tensile strain, but only for elastic strains without considering defect formation and plasticity. Wang et al.^19^ studied the effect of nanoindentation on the local magnetism around the indentation pit, but only for zero spin temperature, i.e., by ignoring the effects of spin dynamics. Castro et al.^20^ coupled molecular dynamics and micromagnetic simulations to demonstrate how the magnetic properties of Fe change under strain.

There are numerous studies of magnetoelastic effects at low strain^21,22^, including recent spin-lattice simulations^23,24^. However, there are many questions remaining partly due to the difficulty of experimentally quantifying both magnetization and strain at the nanoscale, particularly at large strain values. There are several experiments exploring the role of pressure in magnetization^25^. Fe under pressure has been extensively studied using experiments, models and simulations^26–32^. It is known that the the bcc $\rightarrow$ hcp transformation involves both a volume collapse and a ferromagnetic to non-magnetic transition^33,34^, and therefore the need for a concurrent treatment of magnetic and structural order has been pointed out^35^. As another example, Kong et al.^36^ found that plasticity in a Ni thin film, in the form of stacking faults, modified magnetic domain recovery under cyclic strain. Coupled spin-lattice dynamics would be required to include such defects, which nucleate and evolve under strain and, therefore, cannot be included in micromagnetic simulations, nor in atomistic spin dynamics simulations with a fixed lattice.

**Figure 1:**
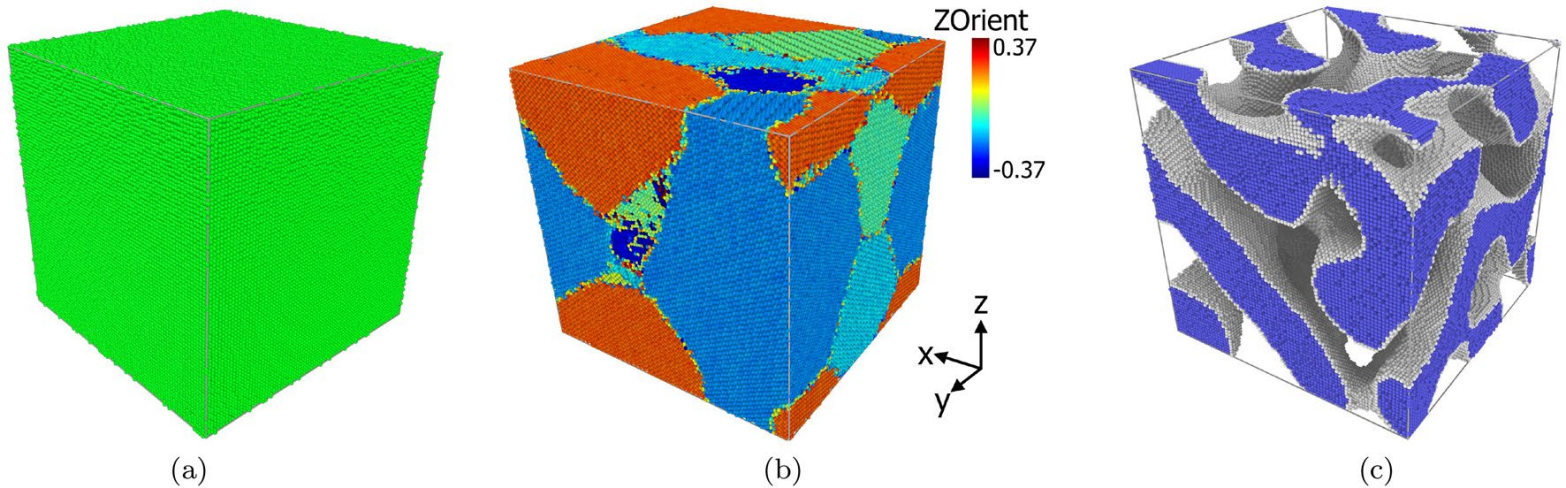
The three Fe samples investigated: (**a**) single-crystal, (**b**) poly-crystal with a color code differentiating individual grains by the orientation towards the *z* axis, (**c**) nanofoam with surface atoms shown in gray and interior atoms in blue.

In this paper, we use spin-lattice dynamics to study the effect of uniaxial compression on three different Fe samples: a single crystal, a polycrystal and a foam structure as illustrated in Fig. 1. Uniaxial rather than hydrostatic compression is modeled since it generates shear strain within the samples that promotes plasticity. We monitor the evolution of pressure during uniaxial compression and correlate it both with the emergence of microstructure (defects) in the samples and with the evolution of the magnetization. This dual consideration of both defects and magnetization allows us to obtain a microscopic understanding of the magnetization changes under compression and plasticity evolution. Our study of the simple ferromagnetic metal, Fe, may prove helpful to understand the causes of the interplay of extended defect structures and magnetism in more complex magnetic materials.

## Methods

### Spin-lattice dynamics

Spin-lattice dynamics simulations^13,37^ combine classical molecular dynamics simulations with the dynamics of the spin system. Molecular dynamics is used to evaluate the motion of the lattice atoms—in the simplest case the thermal motion, but also the motion under external forces —while the dynamics of the spin system is based on the stochastic Landau-Lifshitz (sLL) equation.

For Fe, we use the interatomic interaction potential by Chamati et al.^38^ which is of the embedded-atom-model type. This potential describes well several properties of Fe and was employed in our previous SLD simulations^37,39^, but is not typically used for simulations at relatively high pressures. We note that the Voter-Chen potential^40^ has been successful in displaying some qualitative results of the bcc to hcp transformation of Fe under pressure, but it has several shortcomings, which can be avoided by using a modified Ackland potential from Gunkelmann et al.^26^. Preliminary results using this latter potential display similar behavior to the Chamati potential reported here.

The motion of atoms is coupled to the motion of spins by two terms: (i) The exchange interaction

1 $$\begin{aligned} H_{\text{ex}}=-J(r_{ij}) ({\varvec{s}}_i \cdot {\varvec{s}}_j -1) \, \end{aligned}$$

and (ii) the cubic magnetic anisotropy

2 $$\begin{aligned} \begin{aligned} H_{\text{cubic}}&= \sum _{i=1}^{N} K_1 [ \left( \varvec{s}_i \cdot \varvec{n}_1\right) ^2 \left( \varvec{s}_i \cdot \varvec{n}_2\right) ^2 + \left( \varvec{s}_i \cdot \varvec{n}_2\right) ^2 \left( \varvec{s}_i \cdot \varvec{n}_3\right) ^2 + \left( \varvec{s}_i \cdot \varvec{n}_1\right) ^2 \left( \varvec{s}_i \cdot \varvec{n}_3\right) ^2 ] \\&\quad + K_2 \left( \varvec{s}_i \cdot \varvec{n}_1\right) ^2 \left( \varvec{s}_i \cdot \varvec{n}_2\right) ^2 \left( \varvec{s}_i \cdot \varvec{n}_3\right) ^2\, . \end{aligned} \end{aligned}$$

Here, $\varvec{s}_i$ is the unit spin vector of atom *i* and *N* the total number of atomistic spins in the system. In atomistic spin dynamics or spin-lattice dynamics there is no explicit demagnetization field. This field appears in the micromagnetic simulations as a result of the microscopic dipolar interactions^41^. In addition, dipole-dipole interactions are generally neglected in atomistic spin dynamics simulations because they are more than one order of magnitude smaller than exchange interactions for small single-domain nanostructures, as in the simulations presented here. We checked on these interactions in “Foam”.

In the cubic anisotropy term, Eq. (2), the $\varvec{n}_j$ ($j=1$, 2, 3) are unit vectors along the cubic axes of the crystallite and the anisotropy constants are taken as $K_1=3.5$ eV/atom and $K_2=0.36$ eV/atom^42^. Strain affects anisotropy and more complex functional forms have been applied to simulations with homogeneous pressure. Thus, recent studies^23,24^ used a pressure-dependent Néel-type anisotropy; however, it contributes only of the order of 0.1% relative to exchange. Including realistic anisotropy terms is challenging, since these contributions depend on pressure and temperature^24^, and both evolve strongly with strain in our simulations. Recently, strain anisotropy was found to be crucial in the evolution of domain walls in a micron-scale Ni thin film^36^. In the present study we consider single magnetic domains without external magnetic fields, and the energy associated with the magnetoelastic anisotropy will be much smaller than the exchange energy as mentioned above.

The exchange interaction, Eq. (1), is governed by the exchange parameter $J(r_{ij})$ which depends on the distance, $r_{ij}$, between spins *i* and *j*. The spatial dependence of *J*(*r*) is described by the following Bethe-Slater curve,

3 $$\begin{aligned} J(r_{ij})= & {} 4 \alpha \left( \frac{r_{ij}}{\delta } \right) ^2 \left[ 1-\gamma \left( \frac{r_{ij}}{\delta } \right) ^2 \right] e^{-\left( \frac{r_{ij}}{\delta } \right) ^2} \times \; \Theta (R_c - r_{ij}), \end{aligned}$$

and fitted to data by Ma et al.^43^, cf. Refs.^44^ and^37^, with $\alpha = 96.0$ meV, $\gamma = 0.20$, $\delta = 0.154$ nm. In Eq. (3), $\Theta (R_c - r_{ij})$ is the Heaviside step function and $R_c$ is the cutoff distance. The exchange parameter has a range of $R_c=3.5$ Å, reaching up to the second-nearest neighbor distance of the perfect bcc lattice. We note that the exchange interaction for Fe has been studied repeatedly in the past, but the values depend strongly on the particular approximations used in the calculation^45^, ranging from 13 to 54 meV for the exchange energy between nearest neighbors^46–48^. In most cases, the exchange is nearly zero for neighbors beyond second-nearest neighbors. Our values are those of the parameterization of some ab-initio results by Ma and Dudarev^43^, giving 36 meV for nearest-neighbor interactions. This parameterization leads to excellent agreement with the magnetization versus temperature of bulk Fe^49^. The distance dependence of the exchange interaction will be shown in a figure later in the text.

The magnetization of the sample is defined as

4 $$\begin{aligned} M= \frac{1}{N} \left| \sum _i {\varvec{s}}_i \right| , \end{aligned}$$

such that the magnetization in the ferromagnetic ground state at temperature $T=0$ K amounts to $M=1$ .

The SPIN package of the LAMMPS simulation tool was used to execute all SLD simulations^50,51^. The current version does not allow to include changes in the size of the atomic magnetic moments due to strain; however, we discuss possible effects at the end of “Single-crystalline sample”.

As was mentioned earlier, atomic motion is coupled to spin dynamics, in the sense that the direction of the spins can change due to the lattice vibrations, and the atomic forces depend on the spin rotation as well, see Eq. (6) below. This coupled dynamics is governed by the following Langevin equations^50^,

5 $$\begin{aligned} \frac{d\varvec{r}_i}{dt}= & {} \frac{\varvec{p}_i}{m_i} \,, \end{aligned}$$

6 $$\begin{aligned} \frac{d\varvec{p}_i}{dt}= & {} \sum _{i,j,i\ne j}^{N} \left[ -\frac{dV \left( r_{ij}\right) }{dr_{ij}} + \frac{dJ\left( r_{ij}\right) }{dr_{ij}} \varvec{s}_i \cdot \varvec{s}_j \right] \varvec{e}_{ij} - \frac{\gamma _L}{m_i} \varvec{p}_i + \varvec{\xi }_i \,, \end{aligned}$$

7 $$\begin{aligned} \frac{d\varvec{s}_i}{dt}= & {} \frac{1}{1+\lambda _s^2} \left[ \left( \varvec{\omega _i} + \varvec{\zeta }_i \right) \times \varvec{s}_i + \lambda _s\varvec{s}_i \times \left( \varvec{\omega }_i \times \varvec{s}_i\right) \right] \,. \end{aligned}$$

In Eq. (6), $V(r_{ij})$ is the interatomic potential, $\varvec{e}_{\varvec{ij}}$ is the unit vector connecting atoms *i* and *j*, and $\gamma _L$ is the lattice damping parameter, relating the lattice to an external bath or thermostat. The last term, $\varvec{\xi }(t)$, is a random fluctuating force drawn from a Gaussian distribution with

8 $$\begin{aligned} \langle \varvec{\xi }(t)\rangle= & {} 0 \;, \nonumber \\ \langle \xi _a(t) \xi _b(t') \rangle= & {} 2 D_L \delta _{a b} \delta (t-t') \;, \end{aligned}$$

with *a* and *b* indicating Cartesian vector components, and $D_L$ being the noise amplitude given by $D_L=\gamma _L k_B T$, where *T* is the thermostat temperature and $k_B$ is the Boltzmann constant.

As previously mentioned, the spin dynamics is based on the sLL equation, see Eq. (7). Here, $\varvec{\omega }_i =- \frac{1}{\hbar } \frac{\partial \mathcal {H}_{\text{mag}}}{\partial \varvec{s}_i}$ is the effective field acting on spin *i*, with $\mathcal {H}_{\text{mag}}= H_{\text{ex}} + H_{\text{cubic}}$, see Eqs. (1) and (2). The spin damping parameter $\lambda _s$ is related to the spin-subsystem thermostat and $\varvec{\zeta }(t)$ is the stochastic field which is also drawn from a Gaussian probability distribution with

9 $$\begin{aligned} \langle \varvec{\zeta }(t)\rangle= & {} 0 \;, \nonumber \\ \langle \zeta _a(t) \zeta _b(t') \rangle= & {} 2 D_S \delta _{a b} \delta (t-t') \;, \end{aligned}$$

and the noise amplitude given by

10 $$\begin{aligned} D_S=\frac{\lambda _s (1+\lambda _s^2) k_B T}{\hbar }. \end{aligned}$$

While we use separate Langevin thermostats for the lattice and spin subsystems, both are set to the same temperature and the damping parameters used are $\lambda _s = 0.01$ and $\gamma _L = 1$/ps.

### Sample construction

We employ cubic Fe crystals containing around one million atoms with an edge length of 23.2 nm. Besides the single-crystalline sample, we also generate a polycrystal by using the free software ‘atomsk’^52^. This sample contains 6 grains with an average size of 9.75 nm, cf. Fig. 1b. In order to equilibrate the grain boundaries, the polycrystalline sample is annealed according to the recipe of Ref.^26^ by heating to 1230 K, equilibrating for 100 ps and then cooling down to 300 K.

The nanofoam is created using a numerical method based on the spinodal decomposition of a binary alloy as a model for the bicontinuous microstructure of a nanofoam^53^. The foam structure is illustrated in Fig. 1c. Inside the foam ligaments, the Fe is single-crystalline; i.e., the foam does not contain any grain boundaries by construction. Further details of the construction method are given in Ref.^54^. The porosity of the foam—defined as the ratio of the void volume to the total volume of the foam—amounts to $p=0.5$. Its average ligament diameter is 5 nm; this is also the average diameter of voids in the foam. A further characteristics of a foam is its fraction of surface atoms, $n_s$. For the foam used in our study, it amounts to $n_s=15\%$. After construction, the samples are relaxed for a time period of 50 ps at a temperature of 300 K, with a barostat to obtain zero pressure.

All samples use periodic boundary conditions and are further equilibrated at a temperature of 300 K for 50 ps before starting the compression simulations. The three structures used here are illustrated in Fig. 1.

### Compression

The compression is performed by straining the samples uniaxially with a strain rate of $10^9$ s$^{-1}$. The lateral boundary conditions are chosen such as to keep the stress components perpendicular to the lateral boundaries at zero, in agreement with a compression under uniaxial stress. The positive tensile lateral strain creates a 3D strain state, resulting in very low volumetric strain. The final strain of 20% along the compression direction is thus achieved after 200 ps simulation time. Since the samples are in a ferromagnetic state this is sufficiently slow to allow for spin equilibration during the compression phase^41^. For the bulk single crystal and the foam, the compression is along the [100] direction.

### Analysis of the sample properties and microstructure

OVITO^55^ is employed to render snapshots and to analyze sample microstructure. Dislocation length, $L_{\text{disl}}$, is calculated with the Dislocation Extraction Analysis (DXA) tool within OVITO. Dislocation plasticity is associated with dislocation lines inside grains, and not with possible dislocation structures identified in grain boundaries. To eliminate such grain boundary dislocation networks, only bcc atoms are considered by DXA. This is an approximation that is also applied to the single crystal samples, given the possible transformation into a nanocrystal under pressure. The dislocation density is then calculated as $\rho = L_{\text{disl}}/V$, where *V* is the solid volume of the sample, which is much smaller than the entire simulation volume for the nanofoam samples. Crystal structures are obtained using Polyhedral Template Matching (PTM)^56^, with the parameter RMSD set to 0.2.

The Crystal Analysis Tool (CAT)^57^ is used to identify twin boundaries by matching a bcc-twin pattern to the pattern from adjacent crystallites in the simulations.

### Stress and strain calculation

Compression proceeds along the *x* direction, and lateral stress is kept to zero. This means that, neglecting off-diagonal terms, the stress tensor only has one non-zero component, $P_{xx}$; we will denote this component as the uniaxial stress. Pressure is then $P = P_{xx}/3$, being far from hydrostatic. The von-Mises stress, which provides a measure of shear stress, is $\sigma _{\text{VM}}= P_{xx} = 3 P$. Yield strength and flow stress are given from the corresponding von-Mises stress values, with flow stress taken as the average of $\sigma _{\text{VM}}$ at large strain, between 19 and 20%.

Note that the stress values given by LAMMPS assume that the sample solid volume is equal to the box volume. This is not valid for the nanofoam samples, and stress is therefore scaled by the inverse of solid volume fraction for those cases.

Furthermore, we write strain referring to the strain along the compression direction, and as a positive quantity, even though it is compressive, to ease the description. The volumetric strain is always much smaller than this strain.

## Results

### Single-crystalline sample

**Figure 2:**
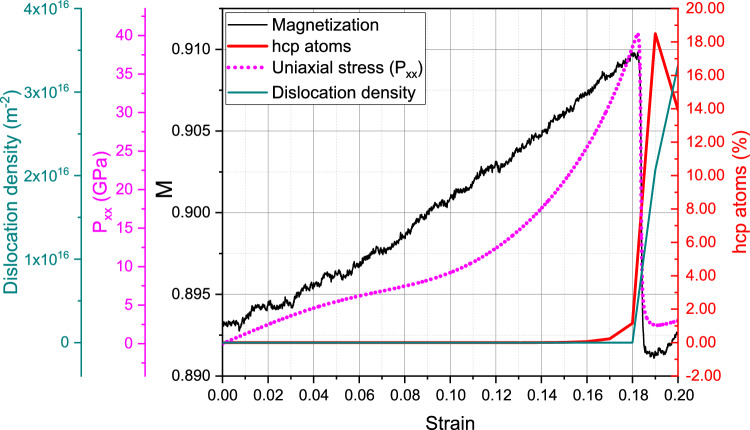
Compression of a single-crystalline sample at 300 K: variation of uniaxial stress, magnetization, dislocation length and hcp atom fraction with strain.

We start with the discussion of the single-crystalline sample at room temperature, 300 K. Figure 2 assembles the important characteristics as a function of strain. Uniaxial stress increases up to strains of around 18% featuring the elastic compression of the defect-free ideal crystal. The volumetric strain increases in the elastic region and reaches a maximum of only 8% at the yield point. Yield strength and flow stress are $\sim$ 39 GPa and $\sim$ 2.5 GPa, respectively. For the perfect crystal this yield is due to the start of the homogeneous bcc $\rightarrow$ hcp phase transformation, which has been extensively studied for uniaxial strain conditions, as mentioned before. For a perfect single crystal, the transition proceeds without any plasticity by dislocations nor twins, and similar elastic limits have been reported for Fe under uniaxial strain and high strain rate^31^. However, triaxial strain is expected to modify the transition pressure compared to uniaxial strain^58^.

The new phase can be considered a ‘defect’ of the original lattice, which can be monitored by the number of hcp atoms. In addition, the boundaries between the bcc and hcp phases include disordered, defective atoms, with unknown structure according to PTM. Here, the fraction of atoms in an hcp environment abruptly increases at the yield point, as expected.

Since the simulation prescribes vanishing lateral stress at the lateral sides of the simulation volume, the phase transformation leads to a rapid lateral volume expansion reducing the volumetric strain nearly to zero. This drives both the pressure and the shear stress down, causing a significant amount of back-transformation to the bcc phase as the strain along the compression direction increases. At 300 K, the phase transformation is only partial, reaching an hcp fraction of nearly 19%.

**Figure 3:**
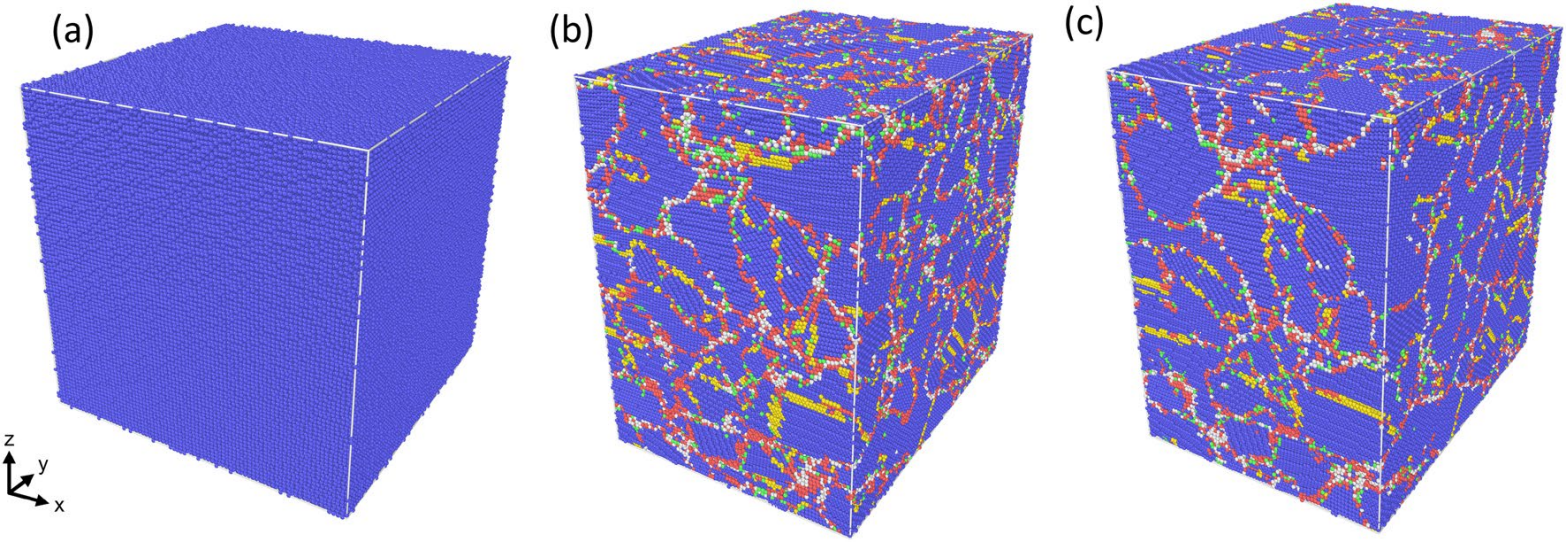
Snapshots showing the microstructure developing in the single-crystalline sample at 900 K at a strain of (**a**) 0%, (**b**) 19%, and (**c**) 20%. Colors denote the local structure: blue-bcc, red-hcp, gree-fcc, yellow-twin boundary.

Because different hcp variants can nucleate nearly simultaneously, several hcp nanoclusters are formed, separated by bcc regions. These clusters will back-transform to bcc nanograins, resulting in a a final nanocrystalline structure as Fig. 3 demonstrates. The grain segmentation tool from OVITO gives a number of 86 grains in the structure at 20% strain. Guo et al.^59^ used the Voter-Chen potential^40^ to compress Fe along [001], finding that uniaxial strain led to nearly complete recovery of the initial Fe single crystal, while quasi-triaxial strain led to a nanocrystal, as observed in our simulations, with a triaxial strain state.

We note that there are dislocations in the bcc phase after the phase transition starts. In the final frame, there is a large dislocation density. There are also a few small twins in the recovered bcc nanograins, as already reported from simulations^26^ and shown in Fig. 3.

Of highest interest is the evolution of the average magnetization, *M*, of the sample during compression. Figure 2 shows that *M* closely mirrors the evolution of the uniaxial stress. However, the change in magnetization during the elastic phase is small, increasing from the equilibrium value of 0.893 to its maximum of 0.910 by only around 2%. This increase is caused by two factors: (i) the average nearest-neighbor distance decreases during compression, increasing the exchange *J*(*r*), and (ii) the average coordination also grows, additionally increasing the exchange energy. Both factors can be glanced from additional information in the Supplementary Material S1.

The phase transition starting close to 18% produces significant disorder, mostly from irregular phase boundaries, but also from dislocation cores in the bcc phase. The material expands and the volumetric strain is reduced to nearly zero, returning to a defective bcc nanocrystal, with nearest-neighbor distance and coordination similar to the unstrained crystal. Therefore, as expected, the magnetization follows the same trend as the pressure, assuming values only very slightly below the initial value of the ideal crystal.

**Figure 4:**
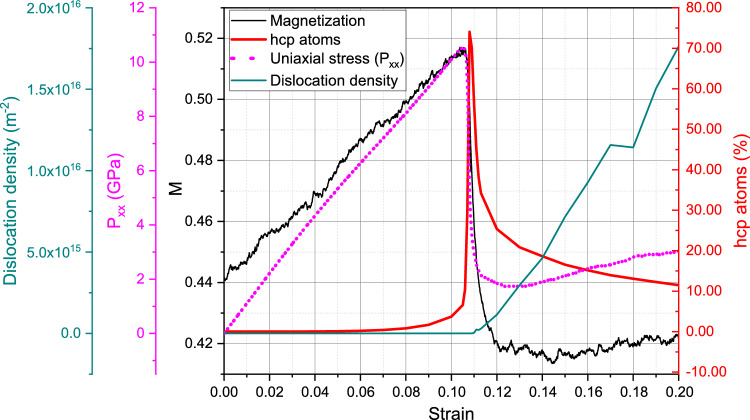
Compression of a single-crystalline sample at 900 K: variation of uniaxial stress, magnetization, dislocation length and hcp atom fraction with strain.

**Figure 5:**
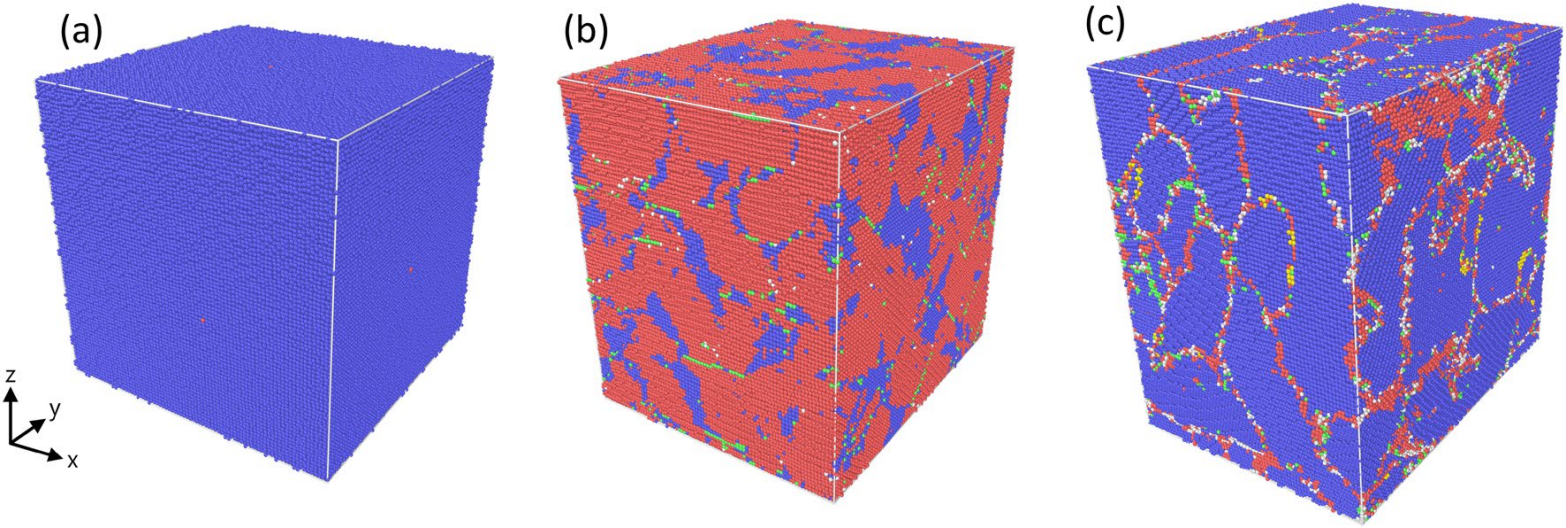
Snapshots showing the microstructure developing in the single-crystalline sample at 900 K at a strain of (**a**) 0%, (**b**) 11%, and (**c**) 20%. Colors denote the local structure as in Fig. 3.

These effects will now be discussed for the compression at 900 K, where the magnetic effects are more pronounced. Here, Fig. 4 assembles the characteristics of the sample under compression and we find good qualitative agreement with the results for 300 K, Fig. 2. The yield stress now occurs for lower strains of around 10.5% at a yield stress of around 10.4 GPa, while the flow stress remains at around 3.0 GPa—similar to the 300-K case. The volumetric strain maximum is only 3% at the yield point and the rapid follow-up volume expansion leads to a volume larger than the initial one. Same as the case for 300 K, the onset of yield is characterized by the solid-state phase transformation of the bcc crystal to hcp, see Fig. 5, where a multitude of hcp grains (62 grains) is visible at 11% strain and about 75% of the sample becomes hcp. Our simulations at larger temperatures show a lower bcc-to-hcp transition pressure, in agreement with experimental and modeling results^60^.

The many different hcp grains available in the hcp phase result in a multitude of bcc grains after the back-transformation; this explains the nanocrystalline structure of the bcc sample at strains of 20% in Fig. 5. The sample also includes some dislocations and nanotwins inside the final bcc grains.

The change in magnetization during the elastic regime is now sizable and amounts to 17%. The reduction of *M* after the end of the elastic phase is again caused by the same factors discussed for 300 K. In the flow stress phase, *M* assumes values below those of the unstrained ideal crystal, due to the many grain boundaries and defects present in the back-transformed material.

**Figure 6:**
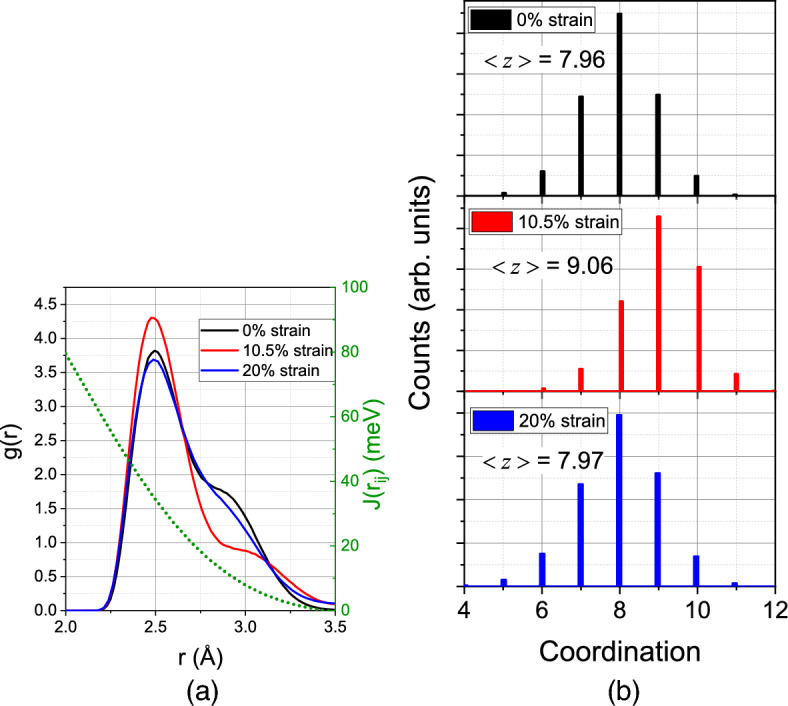
Compression of a single-crystalline sample at 900 K: (**a**) change of the pair distribution function, *g*(*r*), for 0, 10.5 and 20% compressive strain; (**b**) distribution of coordination numbers *z* for several compressive strain values. The green dotted line in (**a**) shows distance dependence of the exchange parameter *J* for comparison.

In order to analyze the magnetization changes more quantitatively, we plot in Fig. 6a the pair correlation function, *g*(*r*), at 900 K. In this figure, a comparison is made between an unstrained crystal and a crystal strained to 10.5%, i.e., around the maximum strain before the transformation starts. At zero strain, the first and second-neighbor positions—at around 2.49 and 2.87 Å, respectively—are strongly broadened due to the high temperature; the third-neighbor position at 4.04 Å is not visible in this plot, since the plot only includes distances up to the cut-off radius of the exchange function, $r_c=3.5$ Å. At a strain of 10.5%, atoms are pushed towards the nearest-neighbor positions. The analysis is further quantified in Fig. 6b, which presents the coordination number *z* of nearest neighbors. It was obtained from the pair correlation, Fig. 6a, by considering all atoms with a distance of < 2.683 Å as nearest neighbors; this distance cutoff gives a symmetric distribution of *z* for zero strain with an average coordination of 7.96, close to the ideal 8 of the bcc crystal at low temperature. At 10.5% strain, the distribution has shifted towards higher coordination with an average of $\langle z \rangle =9.06$. The third panel at the final compressive strain of 20% gives again a reduced coordination close to the bcc value, since disorder from dislocation cores and grain boundaries is similar to the disorder produced by the high temperature.

The analysis of the mean coordination $\langle z \rangle$ is useful since the critical temperature of the ferromagnetic-paramagnetic phase transition, $T_c$, depends on it. The Ising model simply obtains $k_BT_c=\langle z \rangle J$, where $k_B$ is Boltzmann’s constant, and *J* is the exchange interaction for nearest-neighbor sites. More refined models of ferromagnetism keep the proportionality of $T_c$ with *J* and $\langle z \rangle$^61^. Note that Fig. 6a shows that, for $T=900$ K, nearest-neighbor distances hardly change during the compression such that it appears appropriate to assume *J* unchanged during the compression. An increase of $\langle z \rangle$ by almost 14% can thus be interpreted as an increase of $T_c$ by the same fraction.

The increase of $T_c$ with pressure during the elastic phase corresponds to the decrease of $T_c$ during tensile expansion of Fe that was discussed in previous work using ab-initio calculations^18^. The decrease of $T_c$ observed due to defect formation was discussed previously using SLD simulations of vacancy-loaded crystals^39^.

The effect of compressive strain on the magnetism of Fe is qualitatively similar, but quantitatively considerably stronger, at 900 K than at 300 K. The reason hereto is that 900 K is close to the critical temperature, $T_c$, of our system, which amounts to 966 K for the interatomic potential and the spin Hamiltonian chosen^39,54^. Close to the critical temperature, changes in atomic coordination affect the correlation of magnetic moments in neighboring atoms more profoundly than at low temperatures.

It may be noted that the addition of a small number of defects to the single-crystalline sample does not change the results qualitatively. We demonstrate this feature for a representative case in the Supplementary Material S1, where a small number of dislocation loops were introduced into the single crystal. The linear increase of the magnetism with uniaxial stress in the elastic compression phase, and the break-down of the magnetization as soon as massive defect formation sets in caused by material yield occurs in close agreement with the results for the single crystal discussed above. Of course, material yield occurs at a smaller strain and the yield stress is shifted to smaller values since dislocations are more easily nucleated in a defective crystal than in an ideal crystal. Dislocation nucleation relaxes the material, reducing pressure and impeding the phase transition for this case.

Besides the spin orientation, which is measured in the average magnetization *M*, also the size of the magnetic moment $\mu$ will change during compression experiments. This is caused by the so-called magneto-volume effect which anti-correlates atomic volume and atomic magnetic moment^1,62,63^. This effect explains the increase of the magnetic moment at surfaces, grain boundaries, vacancies and other defects in iron^8,64^. It has been used to correlate quantitatively the increase in $\mu$ with the defect densities present in an Fe sample^65^. For a dislocation density of $10^{17}$ m$^{-2}$, Ref.^65^ predicts $\mu$ to increase by 4 per mille; this effect is negligible for the 900-K sample, but may interfere with our results on the 300-K sample. In the latter case, it will act to *increase* the magnetization and thus counteracts the decrease of *M* caused by the decreased average coordination $\langle z \rangle$ after the pressure drop. We note that recently another approach to calculating changes in the magnitude of the magnetic moment in spin-lattice dynamics was explored^66,67^.

Our simulations are in line with experiments for Fe$_{92}$Ni$_{08}$ that show an increase in magnetic moment with pressure^25^, until the hcp transition is reached and causes a decrease with pressure. Our results displaying a lower transition pressure at high temperature are also consistent with the finite-temperature magnetic disorder lowering transition barriers^35^.

Atomic magnetic moments depend on local electronic density^64^. This has also been expressed as a dependence on atomic pressure or atomic volume, where lower volume leads to lower magnetic moment^1^, and the volume collapse of the bcc structure leads to an hcp structure with zero magnetic moments^33^. In our simulations, the atomic volume remains similar to the volume at ambient pressure despite the applied uniaxial stress, even across the phase transition. Therefore, if one considers only a simple dependence of the magnetic moment on atomic volume, an approximately constant magnetic moment would be justified during the mechanical deformation. However, ab-initio simulations are needed to assess the magnetic moment in the obtained hcp phase.

### Poly-crystalline sample

While the mechanisms of plasticity and defect creation are more complex in polycrystals than in single crystals, since grain boundaries contribute, the effects on magnetization are similar. We shall focus here on the latter aspect; for a more detailed discussion of the defects formed in the polycrystal under compression in these simulations, we refer the reader to the Supplementary Material S1. We note, however, that the identification of dislocations has to be done with care in order not to include grain-boundary dislocations in the analysis. Also the number of atoms identified as hcp atoms by PTM, which was zero for unstrained single-crystalline samples, now starts at relatively high values; these are an indicator of the grain boundaries existing in the polycrystalline samples.

**Figure 7:**
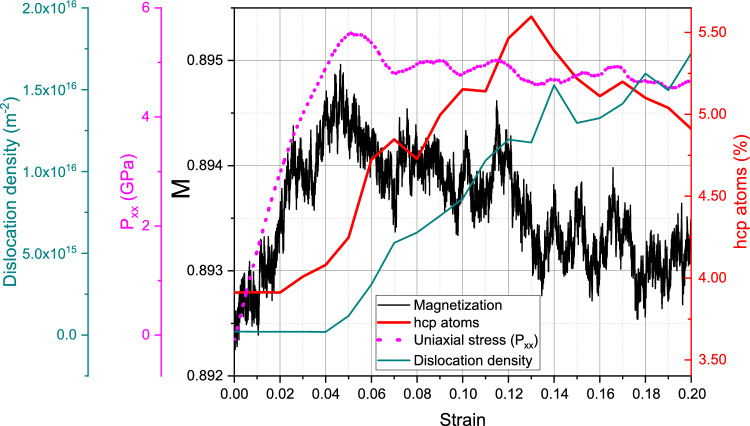
Compression of a poly-crystalline sample at 300 K: variation of uniaxial stress, magnetization, dislocation length and hcp atom fraction with strain.

**Figure 8:**
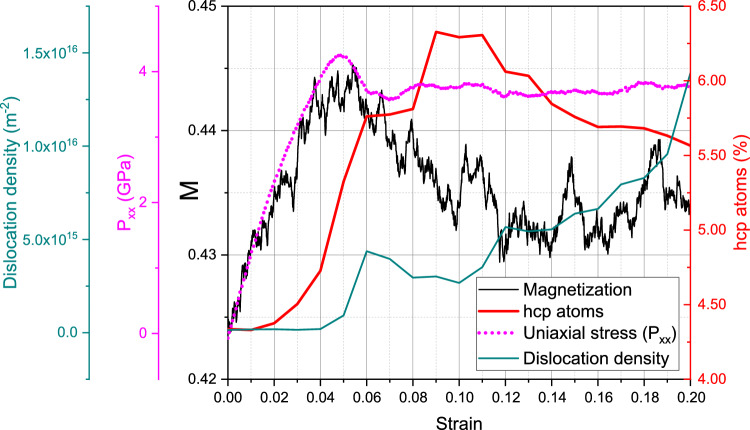
Compression of a poly-crystalline sample at 900 K: variation of uniaxial stress, magnetization, dislocation length and hcp atom fraction with strain.

Figure 7 shows the results at room temperature, 300 K, and Fig. 8 for 900 K; we can discuss these two samples simultaneously. The uniaxial stress increases roughly linearly with strain up to a strain of 5%, when volumetric strain reaches a maximum of about 1%. This regime is not completely ‘elastic’ though, since there is grain boundary activity, and some defects are generated even at such moderate strains as the (slight) increase in the fraction of hcp atoms shows; the creation of dislocations is, however, negligible, in this regime. The magnetization starts at somewhat smaller values than in the single-crystal samples discussed above, at 0.8925 for 300 K and 0.42 at 900 K compared to 0.893 and 0.44 for the single crystals, respectively. These lower values are caused by the defects (viz. grain boundaries) present in the structures, which decrease the effective nearest-neighbor coordination. The final magnetizations reached at the end of the elastic part of the compression phase are even considerably smaller than their single-crystal counterparts; this is primarily caused by the lower strains at which these elastic parts end. However, also the slope of the magnetization increase with strain is smaller than for the single crystals; we assume that this is caused by the grain boundaries which are present in the polycrystalline samples.

The flow stresses reached after the end of the plastic phase amount to 4.5 and 3.5 GPa for 300 and 900 K, respectively. In this plastic phase, dislocations build up continuously and reach densities that are considerably smaller than in the defective single-crystalline samples. Magnetization decreases during the flow phase, similar to the single-crystalline samples; however, the decrease is not as abrupt, since defects were already present from the start of the compression and did not form instantaneously at the end of the elastic phase. Thus the 300-K polycrystalline sample, Fig. 7 shows a rather monotonic decline throughout the flow phase, which agrees nicely with the continuous growth of both dislocations and hcp atoms. The magnetization for the 900-K sample, Fig. 8 reaches a steady value towards the end of the flow phase, which may be explained by the interplay of the growth of dislocation density counteracted by the decrease in hcp atom fraction.

We conclude that the dependence of magnetization on strain is obscured in nano polycrystalline samples due to the presence of defects (grain boundaries) from the beginning of the compression. The ratio between GB atoms and atoms inside grains is, roughly, inversely proportional to grain size, and samples with much larger grains might display a larger magnetic effect under compression.

### Foam

**Figure 9:**
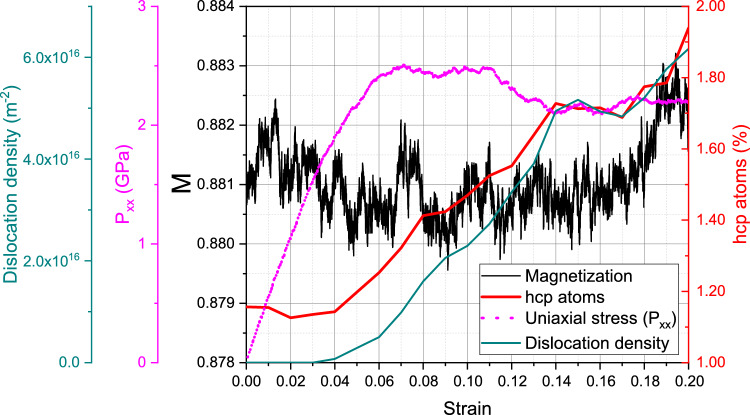
Compression of a foam sample at 300 K: variation of uniaxial stress, magnetization, dislocation length and hcp atom fraction with strain.

**Figure 10:**
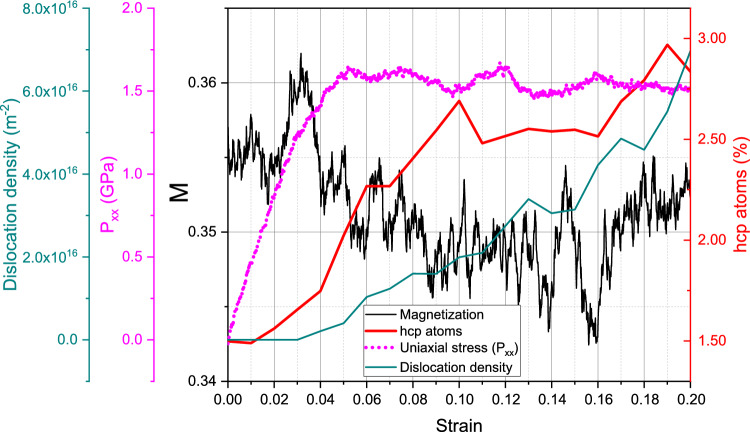
Compression of a foam sample at 900 K: variation of uniaxial stress, magnetization, dislocation length and hcp atom fraction with strain.

As a third system, in which the interplay of compressive strain and magnetization can be discussed, we show the mechanical and magnetic characteristics of a nanofoam at 300 K and 900 K in Figs. 9 and 10, respectively. It is known^54^ that the Curie temperature in foams is reduced with respect to that in bulk Fe. In the foam considered here $T_c$ is around 935 K^54^; our simulation is hence still in the ferromagnetic state of Fe.

Similar to the polycrystals, also the foams show a non-vanishing number of hcp atoms in their unstrained state; these are in reality surface atoms of the ligaments which are wrongly assigned by the PTM crystal analysis tool. During compression, the hcp fraction increases, indicating the beginning of defect formation.

The stress-strain curve shows the typical behavior in nanofoams, with a roughly linear elastic region followed by a stress plateau due to plasticity^68^. The uniaxial stress reaches maximum values of 2.5 (1.6) GPa for the 300 (900) K sample, see Figs. 9 and  10, higher than in the polycrystalline sample. This is caused by the fact that compression not only proceeds by—elastic or plastic—deformation of the ligaments but also by incremental void closure and ligament bending. Porosity decreases from 0.50 initially to 0.43 at 20% strain. Voids change shape and reduce their volume. Considering only the solid volume of the foam, there is an initially growing volumetric strain in the elastic region, reaching 5 (2.5)% at 300 (900) K. In the plastic region, the volume expands, reaching values larger than the initial volume.

The density of dislocations is similar to that found in the defective single crystals and definitely larger than that in the polycrystals, in particular at the higher temperature. The high dislocation density is caused by the fact that the ligament surfaces act as dislocation sources rather than sinks; this is in agreement with previous work on the nanoindentation of Au nanofoams^69^ and also applies to bcc nanofoams^70^. Dislocation absorption at the ligament surfaces will also occur but on time scales larger than our simulation times. Note also that the Fe material in the foam is single-crystalline such that no grain boundaries are present that could absorb dislocations. For a more detailed discussion of the defects formed in the foam under compression, see Supplementary Material S1.

The magnetization in the unstrained state at 900 K is considerably smaller in the foam ($M=0.33$) than in the polycrystal (0.42) or even the single crystal (0.44). This is due to the reduction of the Curie temperature in the foam noted above. During compression, the magnetization does not exhibit any marked changes and the evolution with strain appears to be governed by noise (thermal fluctuations) rather than by the compression.

The nanofoam structure gives a good opportunity to check on the influence of dipole-dipole interactions in our simulations as they may couple the fields of neighboring ligaments. In order to obtain an estimate of the contribution of dipolar interactions, we compare the potential energy of a given spin configuration at a certain strain with and without a dipolar Hamiltonian^50^. The largest contributions are expected for the bicontinuous foam sample, with both filaments and void size around 5 nm. For computational purposes, we only consider spins within a 8 nm radius, which is large enough to include entire filaments and also surfaces across voids. We checked that some variation of this cut-off does not modify significantly the magnetic energy. At 300 K, the change in energy due to dipolar interactions is only 0.0008 meV/atom at 0% strain, and 0.0005 meV/atom at 19% strain. This is less than $3\cdot 10^{-5}$ of the magnetic exchange energy per atom, justifying a posteriori the exclusion of dipolar interactions for the simulations in this work.

We conclude that—in contrast to compact samples—the magnetization in foams is only little affected by compression, which mainly leads to partial void closure and bending of the ligaments. On the other hand, magnetization is based on the (less affected) material in the ligaments. It may be noted that foams exhibit a strong compression/tension asymmetry^68^ such that a tension experiment might give a different picture.

## Summary

Compression of a magnetic material leads to a change in its magnetic properties. We examined the microscopic physics behind this connection between structural and magnetic properties for the special case of bcc-Fe, using both single- and polycrystalline Fe and additionally a nanofoam structure. Our main findings are as follows.

1. During the elastic phase of compression, the magnetization increases. This is caused by a higher population of the nearest-neighbor shell of atoms leading to a higher exchange interaction of spins.
2. In the plastic phase of compression, defects are created. Defects are related to disorder, and include dislocations, grain boundaries and nucleation of a different phase. Concomitantly, the magnetization sinks, as the atomic coordination typically decreases in the defective regions.
3. Both elastic and plastic effects on the magnetization are considerably more pronounced close to the Curie temperature—where the magnetization change can exceed 10%—than at room temperature, where it is only of the order of 2%.
4. Also, the effects are more pronounced in single-crystals than in polycrystals with nanograins. In the latter, the presence of defects in the form of grain boundaries tends to counteract the magnetization increase during the elastic phase of compression. This might change for much larger grain sizes.
5. The effect of compression on nanofoams is minor since compression proceeds mainly by porosity reduction and filament bending—with negligible effect on magnetization—rather than by ligament strain, below the strain where large compaction is achieved.

As our major result, we thus state that it is possible to tailor magnetization by introducing plasticity into the sample. Such a finding has already been observed previously in experiments on Heusler alloys and motivated the idea that dislocation networks can be used to control magnetic properties^71^. On the other hand, in high-entropy alloys of the twinning-induced plasticity (TWIP) and transformation-induced plasticity (TRIP) types, magnetic ordering has been found to influence deformation modes^15^. Analogously, Mu et al.^72^ found that pressure influences the magnetization in a medium-entropy alloy. Pressure loading and unloading modifies magnetic remanence and may help to tailor hysteresis loop magnitudes^25^. Understanding these modifications could help with the analysis of meteoritic FeNi samples, since the remanent magnetization, employed to understand their evolution, is affected by pressure-induced defects^25^. In addition, understanding the role of spins during the plastic deformation of metals is required to study the role of electromagnetic fields in modifying mechanical properties as it was found experimentally^73,74^ and attributed to the interaction of dislocations whose cores contain atoms with different magnetic moments^75^.

We note that micromagnetic simulations^76,77^ have cell sizes that are typically larger than 2 nm, and grain boundaries are described as soft magnets, with low exchange and zero anisotropy, with the exchange used as a fit parameter^78–80^. Grain boundary thicknesses of 4–12 nm are used, due to the spatial resolution inherent to the method. This differs significantly from typical atomistic polycrystal samples, where grain boundaries are at most $\sim$ 1 nm thick, even for complex materials. Our nanocrystal description includes such thin grain boundaries and there is no need to re-fit the lower exchange, which occurs naturally due to the grain boundary disorder, although other factors such as atomic volume might also modify this effective exchange. Of course, our simulations can only handle nanoscale grains with diameters $\mathcal {O}$(10 nm), while micromagnetic simulations can handle grains with sizes $\mathcal {O}$(100 nm).

In contrast to dense samples, magnetic nanofoams can be used if stability of the magnetization under plastic deformation is a required feature.

In future work, it might be most important to include defect-induced changes of the size of the magnetic moment into the calculation. Also, the consideration of magnetic materials other than pure iron will be relevant, in particular alloys.

### Supplementary Information

Supplementary Information.

## Data Availability

All data used for this study are contained in this article.
