# Supplementary material for: Spin-lattice-dynamics analysis of magnetic properties of iron under compression
Source: Sci Rep. 2023 Aug 31;13:14282. doi: 10.1038/s41598-023-41499-2 (PMC10471586; doi:10.1038/s41598-023-41499-2)
Supplement: Supplementary file 1 — Supplementary Information. [file 41598_2023_41499_MOESM1_ESM.pdf]

Spin-lattice-dynamics analysis of magnetic properties of iron under compression
Supplementary Material

Gonzalo dos Santos,[1] Robert Meyer,[2] Diego Tramontina,[1] Eduardo M. Bringa,[1,3] and Herbert M. Urbassek[2,*]

[1]*CONICET and Facultad de Ingeniería, Universidad de Mendoza, Mendoza, 5500 Argentina*
[2]*Physics Department and Research Center OPTIMAS, University Kaiserslautern-Landau, Erwin-Schrödinger-Straße, D-67663 Kaiserslautern, Germany*
[3]*Centro de Nanotecnología Aplicada, Facultad de Ciencias, Universidad Mayor, Santiago, Chile 8580745*
(Dated: July 28, 2023)

This Supplementary Material presents results for a defective single-crystalline sample and further discusses the defect microstructure formed in the single-crystalline, polycrstalline and foam samples.

Single crystals

Fig. S1 includes both the pair correlation function and coordination histograms for various strains. The compression along one direction shifts the nearest neighbor-peak towards a lower value, while the lateral expansion shifts the second nearest neighbors peak towards larger values. This deformation of the bcc cubic cell paves the way for the transition to hcp.

Fig. S2 shows the microstructure and grain orientations developing in the single-crystalline sample at 20 % strain. In this figure, atoms are colored according to misorientation and the twin boundaries are colored in red. This figure highlights the polycrystalline nature of the highly strained sample.

Defective single crystals

A single-crystalline sample containing defects – mainly dislocation loops – is shown in Fig. S3. Fig. S4 assembles the important characteristics – magnetization, uniaxial stress, dislocation length, and hcp fraction – as a function of strain at a temperature of 300 K. The uniaxial stress linearly increases up to strains of around 12 % featuring the elastic compression of the defect-free ideal crystal. The yield strength amounts to around 11 GPa; after yield the flow stress reaches a value of $\sim$ 2.8 GPa.

Defects are easiest monitored by atoms in a non-bcc local crystalline environment. Here, we use the fraction of atoms in an hcp environment to monitor defect creation; this fraction abruptly increases at the yield point. These hcp atoms are indicative of twin boundaries that formed in the crystal, cf. Fig. S5a. Later, dislocations are created continuously from these twin boundaries in the material, such that the length of dislocation lines, $L_{\rm disl}$, monotonically increases. Fig. S5b displays the dislocation network that has formed at the maximum strain, 20 %. The dislocation density is then calculated as $\rho = L_{\rm disl}/V$, where V is the solid volume of the sample. The values obtained correspond to a highly dislocated crystal; Fig. S5b illustrates that the dislocation network that has formed is full of junctions originating from dislocation reactions during the compression. These reactions correspond to a work hardening of the sample which shows up as a slight increase in the flow stress in Fig. S4. Of highest interest is the evolution of the average magnetization, M, of the sample during compression. Fig. S4 shows that M closely mirrors the evolution of the uniaxial stress. However, the change in magnetization during the elastic phase is small, increasing from the equilibrium value of 0.893 to its maximum of 0.903 by only around 1 %.

At a temperature of 900 K, the magnetic effects are more pronounced. Here, Fig. S6 assembles the characteristics of the sample under compression and we find good qualitative agreement with the results for 300 K, Fig. S4. The yield stress now occurs for lower strains of around 7.5 % at a yield stress of $\sim$ 6.5 GPa, while the flow stress remains at around 2.8 GPa – similar to the 300-K case. The onset of yield is characterized by the build-up of twin boundaries, Fig. S7a, which dissolve at higher strains to a dislocation network, Fig. S7b. This network shows less complexity compared to the 300-K case, Fig. S5b, and consequently the dislocation density is smaller. Note that for 900 K, no pressure increase during the flow phase is observed, indicating the lack of work hardening; the dislocation density stays constant in the flow phase. The change in magnetization during the elastic regime is now sizable and amounts to $>$ 10 %. The reduction of M after the end of the elastic phase is again caused by the reduced average number of nearest neighbors in the forming defects; however, in the flow phase, M still keeps values above those of the unstrained ideal crystal.

Fig. S8 shows the dislocations produced under 20 % strain in the defective single-crystalline samples at 300 K, Fig. S8 a, and at 900 K, Fig. S8 b. These are the same dislocations as shown in Figs. 3b and 5b; however, they are analyzed with respect to the edge and screw content of the dislocation segments. To this end, we determine the angle θ between the Burgers vector and the dislocation line vector of each dislocation segment; we denote a

---

*Electronic address: `urbassek@rhrk.uni-kl.de`; URL: `http://www.physik.uni-kl.de/urbassek/`

segment as ‘screw’ if the angle is $< 30°$, as ‘edge’ if it is $> 60°$, and as ‘mixed’ otherwise.

The preponderance of screw segments formed is striking, both at 300 K and at 900 K. Since these are less mobile than edge segments, they stabilize the network formed.

Polycrystals

While the homogeneous nucleation of dislocations in a homogeneous single-crystalline sample only occurs at large strains, and requires the temporary formation of twin boundaries as nuclei, dislocations may nucleate in the poly-crystal at grain boundaries, and hence at smaller strains. On the other hand, the smallness of the grains prevents dislocations to grow as large as in the polycrystalline sample; furthermore, they can be absorbed at grain boundaries: These two facts make the grain boundary density considerably smaller than in the single crystal, as was observed in Figs. 2 and 7 and can be seen in the snapshots of Fig. S9a and b.

The majority of dislocations are of the $1/2\langle 111\rangle$ type, since they have the shortest Burgers vector; as Fig. S9a shows, a few $\langle 100\rangle$ dislocations are identified as well, which often form junctions with $1/2\langle 111\rangle$, and can therefore be assumed to have originated from dislocation reactions. Such a mix of these two dislocation types also characterizes the single crystal, Fig. 3; it is typical for dislocation plasticity in bcc samples at high strain rates, such as in nanoindentation [1].

Fig. S9b resolves the dislocation network differentiating between screw and edge segments. As for the single crystals, the dislocations are mainly of screw character.

Fig. S9c gives an example of non-dislocation plasticity that also forms in polycrystals temporarily, albeit at a smaller amount than in single crystals, as they are mainly observed in the larger grains.

Fig. S10 shows the defect microstructure at 900 K. Similar as in the single crystals, Figs. 3 and 5, the number and length of dislocations is educed at this high temperature. Note that in particular the contribution of $\langle 100\rangle$ segments has nearly vanished, Fig. S10a. Also the amount of edge segments is strongly reduced, Fig. S10b.

Foams

The number of dislocations in the ligaments of the strained foam is astonishingly high, see Figs. S11a and b, as was already evident from Fig. 9. Most dislocations span the diameter of the ligaments, often with straight dislocation lines. Again, most dislocations are of the $1/2\langle 111\rangle$ type, Figs. S11a, and have a preference for screw character, Figs. S11b.

Even though ligament diameters are small, around 5 nm, twin boundaries may be created at sufficient strain, as shown in Figs. S11c.

At high temperature, the dislocation density is even higher than at 300 K, cf. Figs. 9 and 10. Fig. S12 demonstrates that this is accompanied by a lengthening of the dislocation lines.

---

[1] Y. Gao, C. J. Ruestes, D. R. Tramontina, and H. M. Urbassek, J. Mech. Phys. Sol. **75**, 58 (2015).

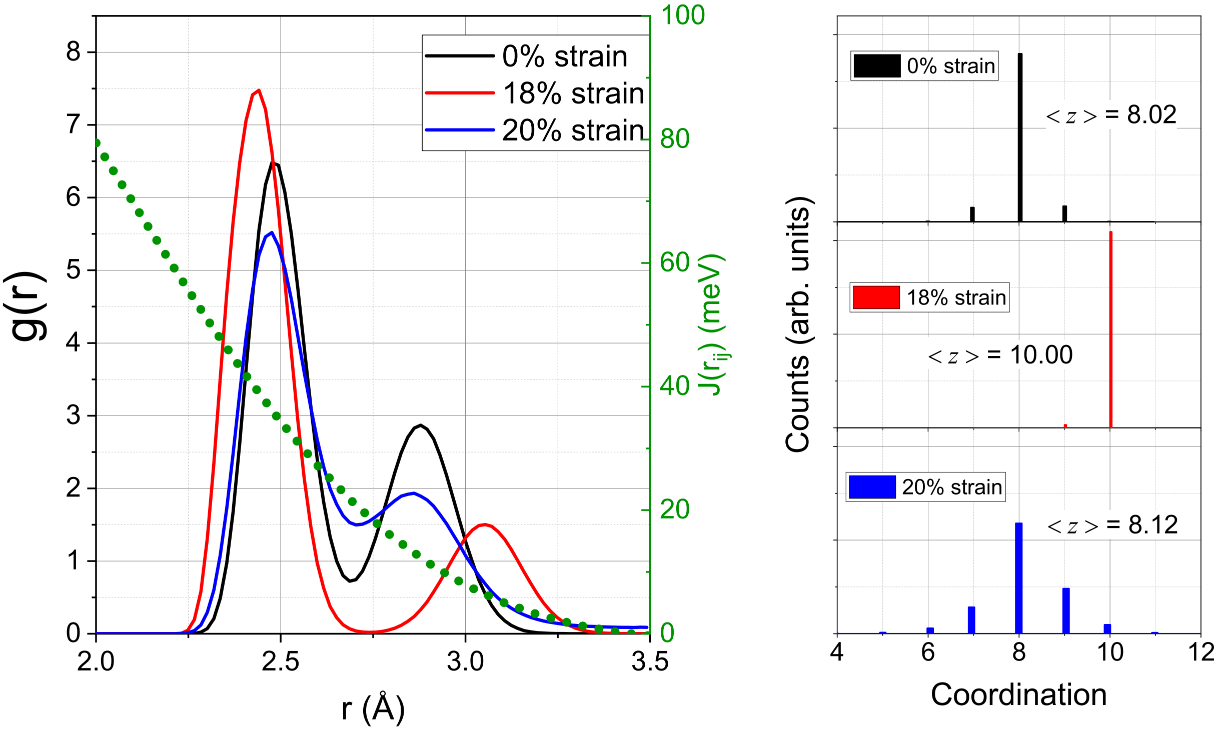

FIG. S1: Compression of a single-crystalline sample at 300 K: (a) change of the pair distribution function, $g(r)$, for 0, 18 and 20 % compressive strain; (b) distribution of coordination numbers z for several compressive strain values. The green dotted line in (a) shows distance dependence of the exchange parameter J for comparison.

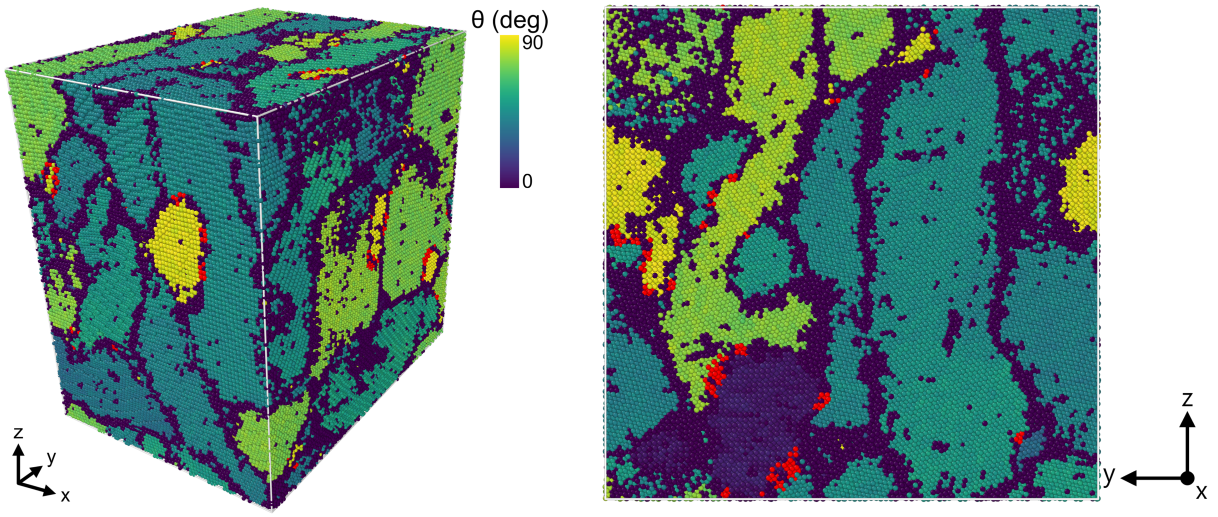

FIG. S2: The single-crystalline sample at 20 % strain and 900 K in a perspective view (left) and a cut (right). Atoms are colored according to their misorientation angle θ in order to highlight the grain structure. Atoms in twin boundaries are colored in red.

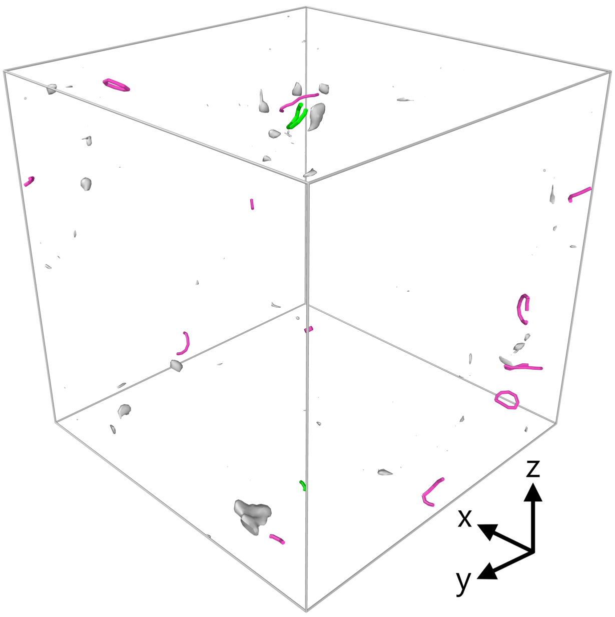

FIG. S3: A defective single crystal. For clarity, only the defects are shown. Vacancy clusters in gray. Color denotes the Burgers vector of the dislocations: 1/2⟨111⟩ (green); ⟨100⟩ (red).

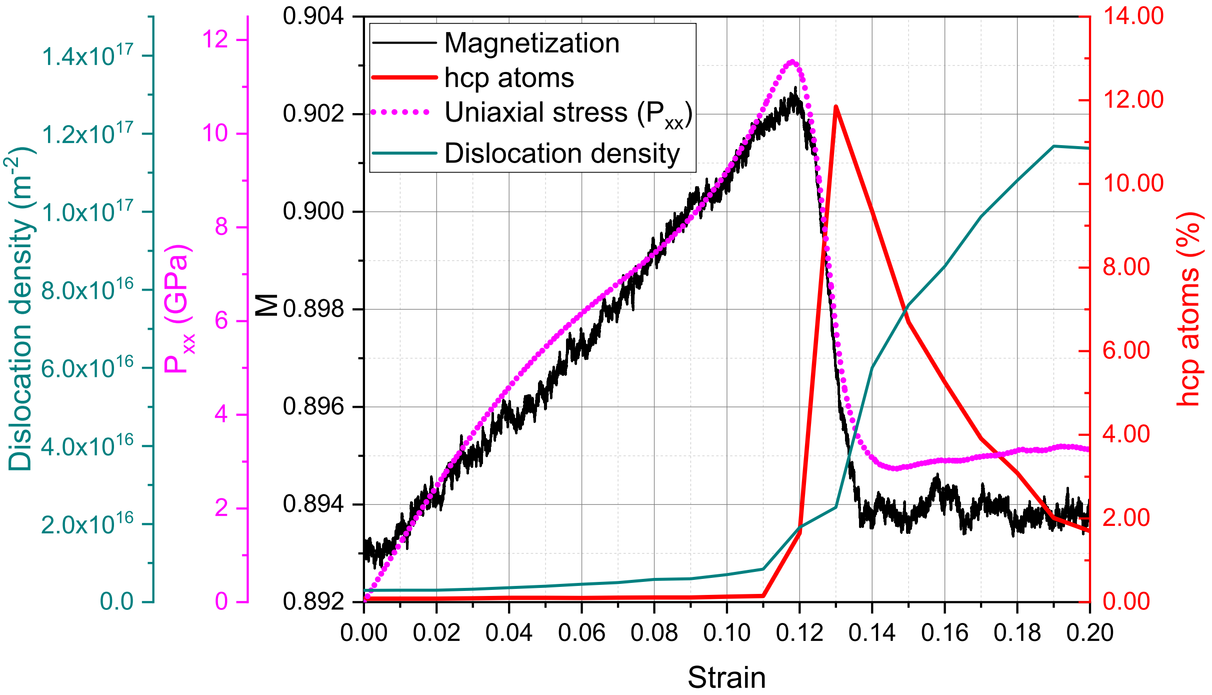

FIG. S4: Compression of a defective single-crystalline sample at 300 K: Variation of uniaxial stress, magnetization, dislocation length and hcp atom fraction with strain.

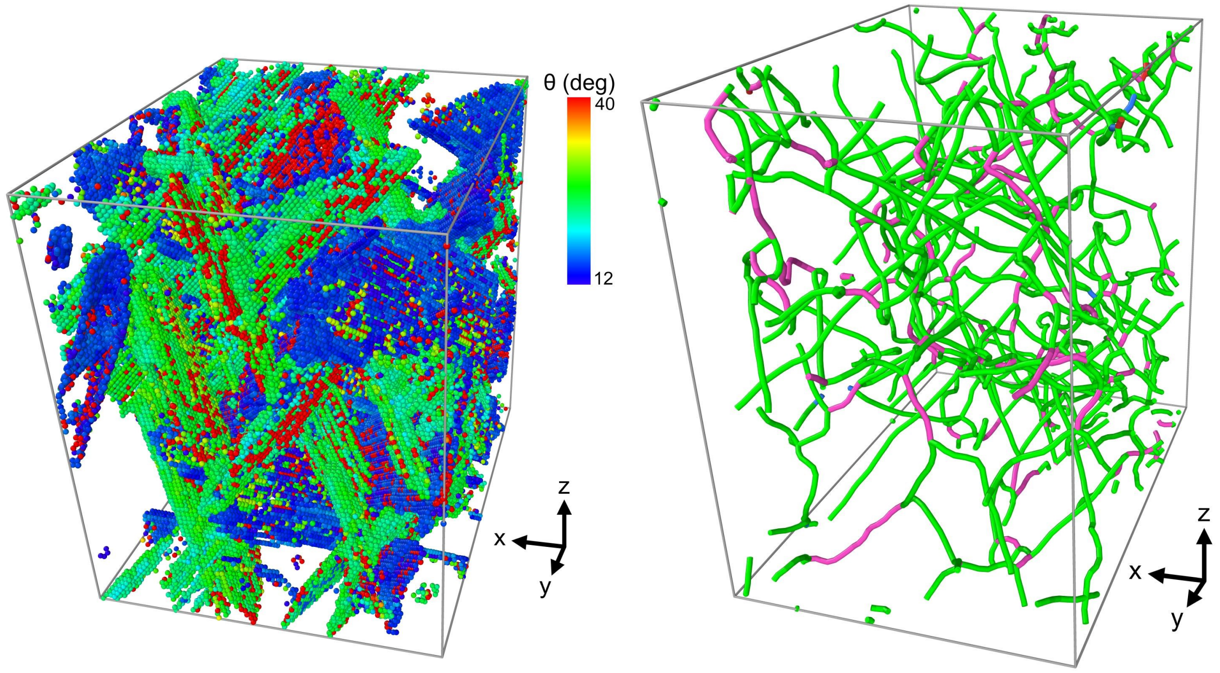

(a)

(b)

FIG. S5: Snapshots showing the microstructure developing in the defective single-crystalline sample at 300 K. (a) Twin boundaries at strain of 13 %. The angle θ used for coloring the structures measures the twin boundary angle towards the [001] crystal direction. (b) Dislocations formed under strain of 20 %. Color denotes the Burgers vector as in Fig. S3.

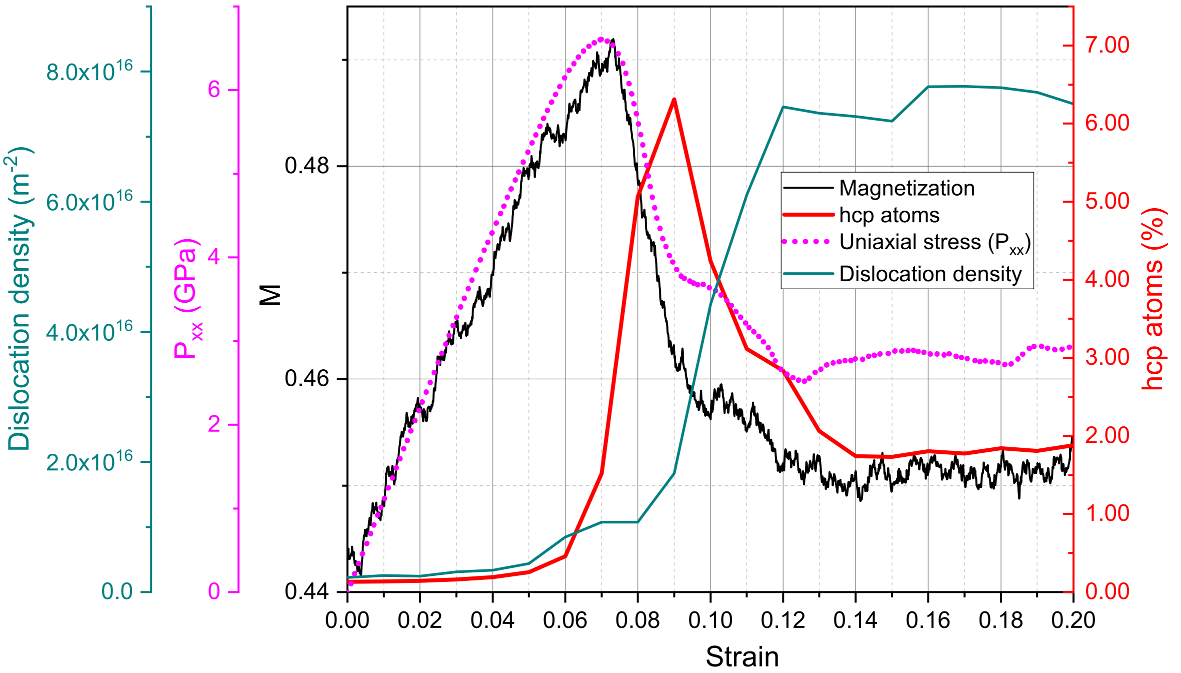

FIG. S6: Compression of a defective single-crystalline sample at 900 K: Variation of uniaxial stress, magnetization, dislocation length and hcp atom fraction with strain.

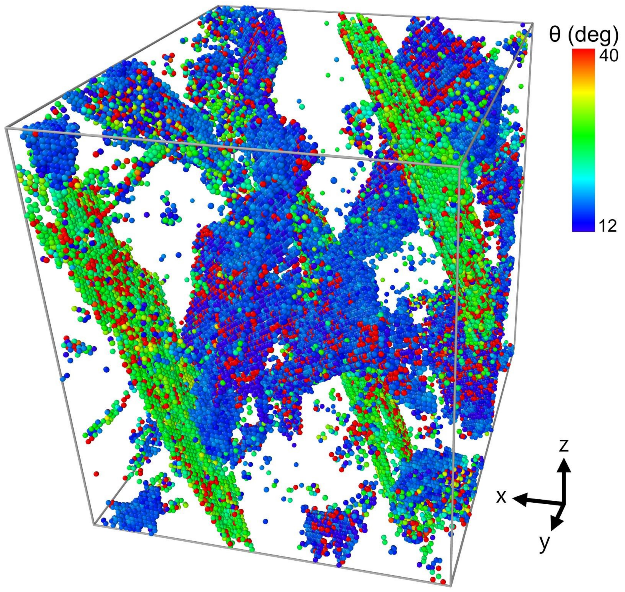

(a)

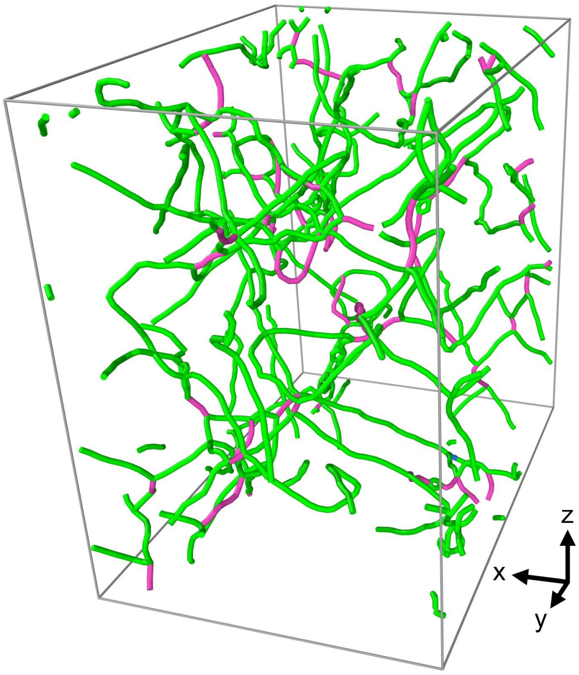

(b)

FIG. S7: Snapshots showing the microstructure developing in the defective single-crystalline sample at 900 K. (a) Twin boundaries and (b) dislocations as in Fig. S3.

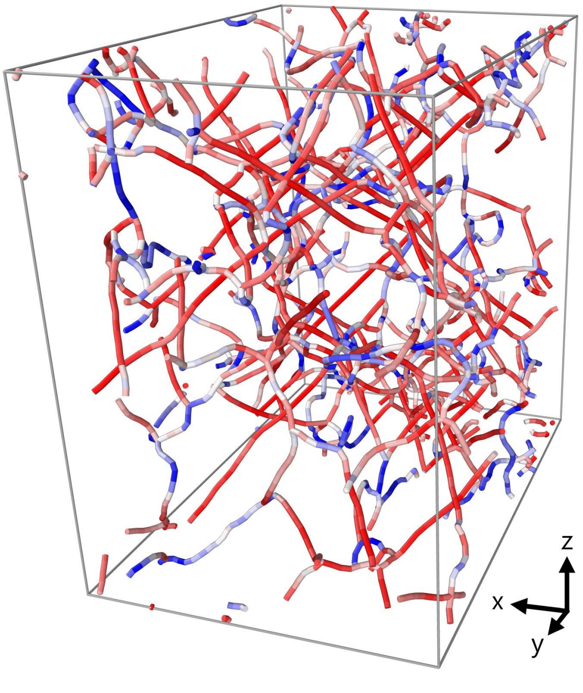

(a)

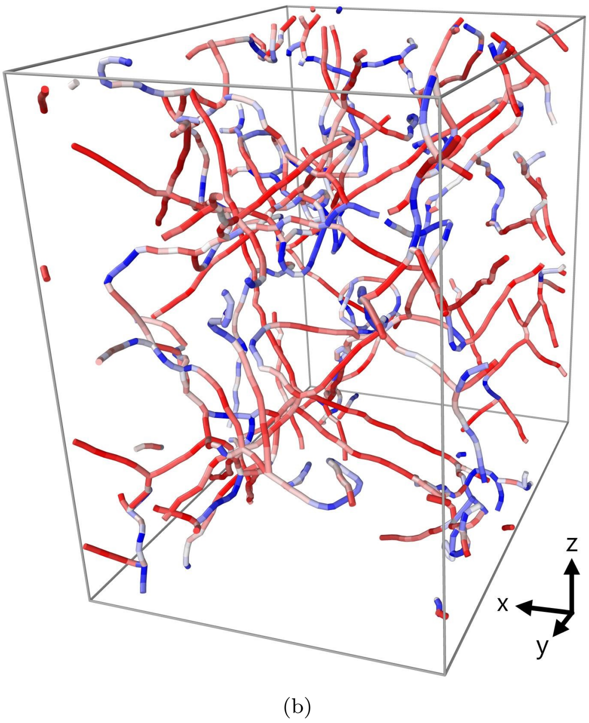

(b)

FIG. S8: Snapshots showing the microstructure developing in the single-crystalline sample at (a) 300 K and at (b) 900 K. Color denotes the screw (red) or edge (blue) character; mixed dislocations are colored gray.

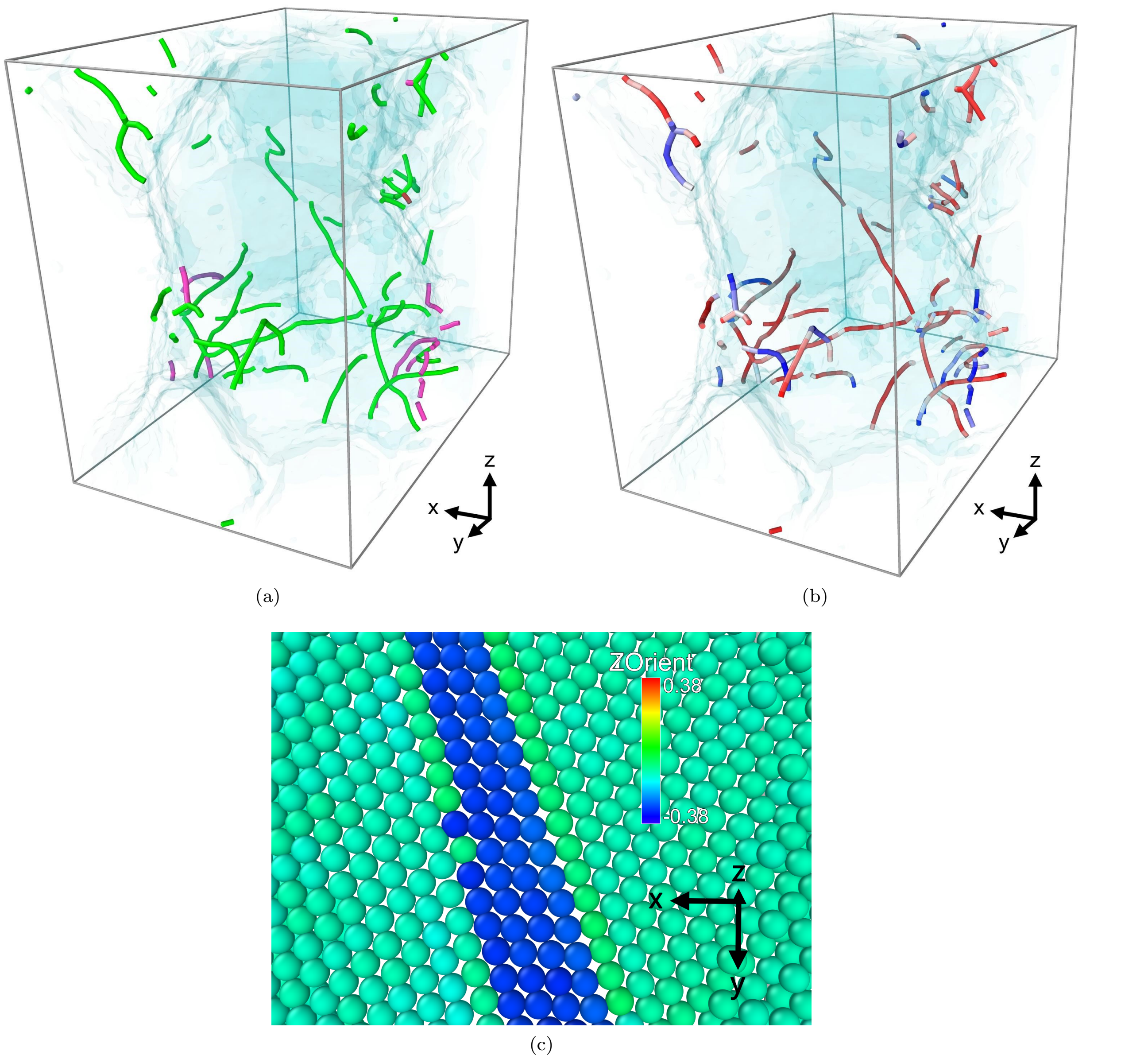

FIG. S9: Snapshots showing the microstructure developing in the polycrystalline sample at 300 K. Dislocations formed under strain of 20 % where color denotes (a) the Burgers vector, as in Fig. 3b, and (b) the screw (red) or edge (blue) character. Shaded structures represent grain boundaries, cf. Fig. 1b. (c) Cross section (depth of 20 Å) at 13 % strain (maximum in hcp atom fraction) showing a twin structure inside a grain; color highlights orientation to z axis.

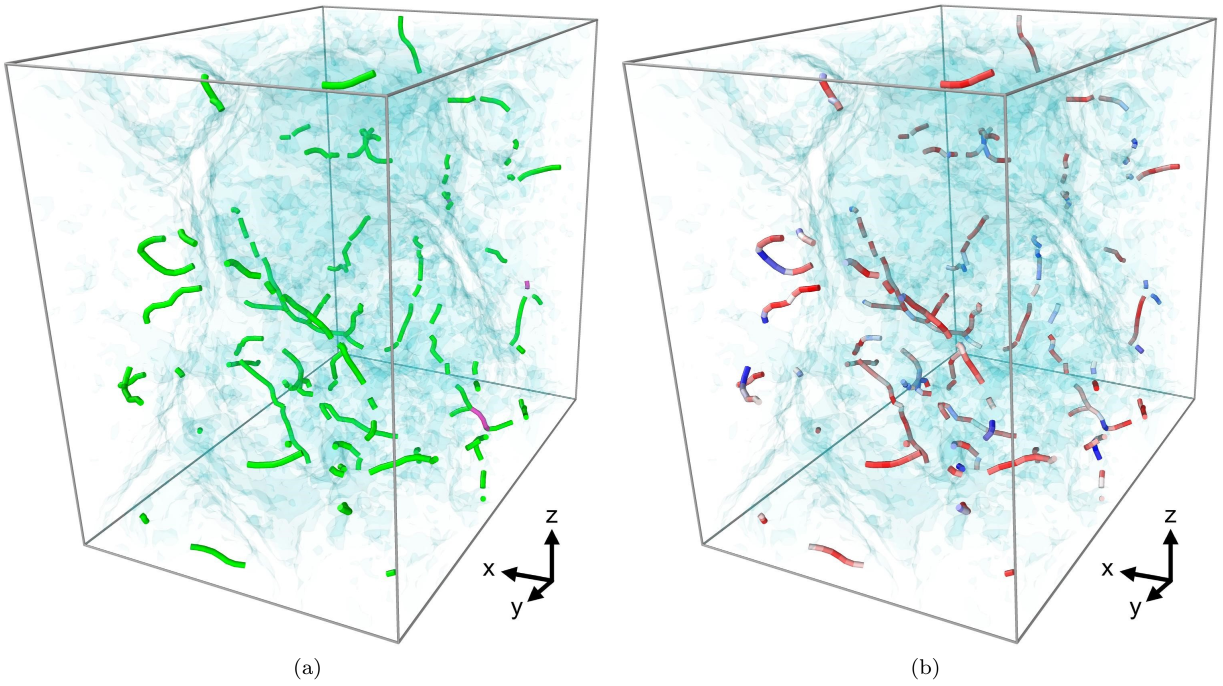

FIG. S10: Snapshots showing the microstructure developing in the polycrystalline sample at 900 K at 20 % strain. Coloring of dislocations as in Figs. S9a and b. Shaded structures represent grain boundaries, cf. Fig. 1b.

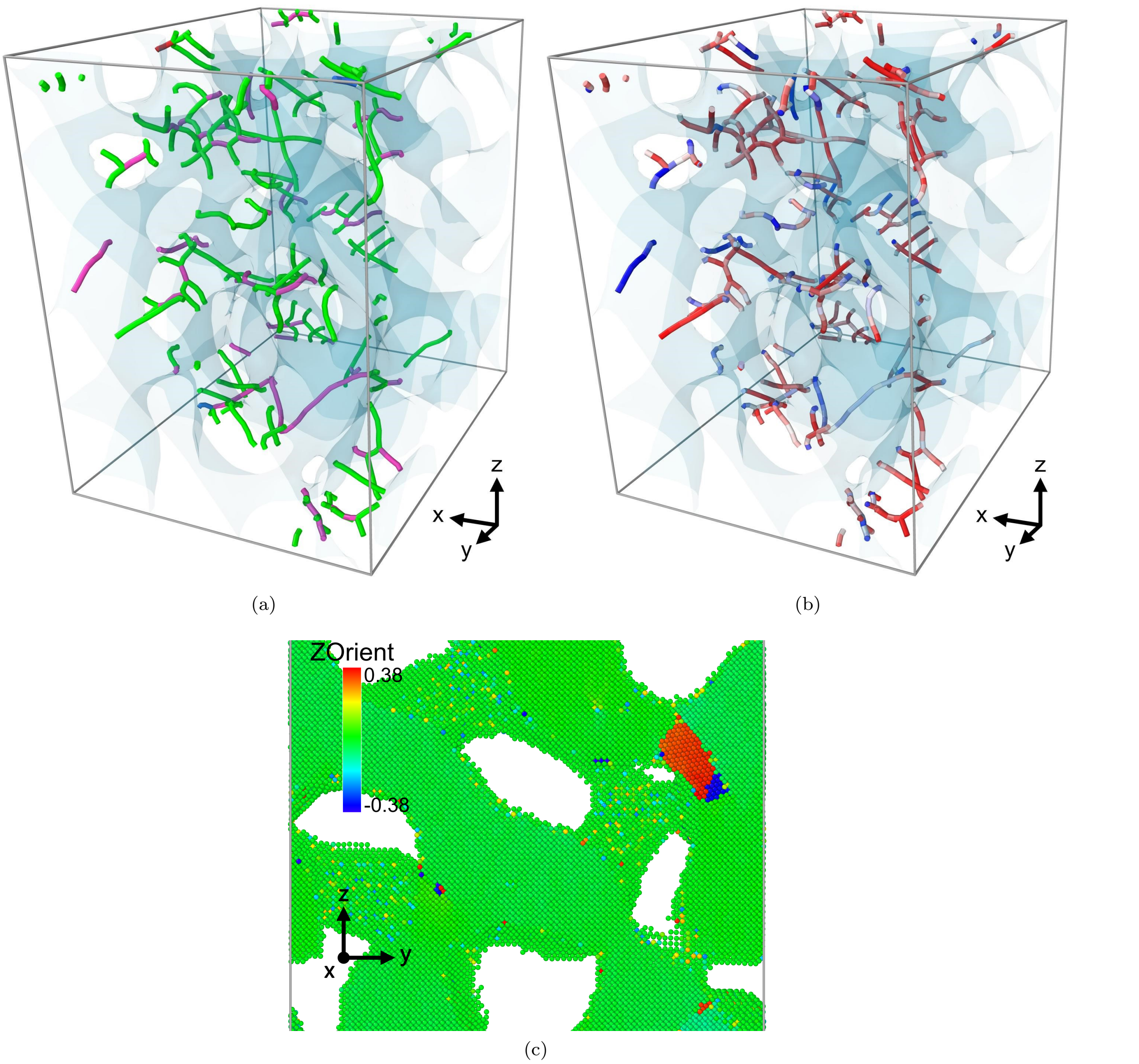

FIG. S11: Snapshots showing the microstructure developing in the nanofoam at 300 K. Dislocations formed under strain of 20 %, where color denotes (a) the Burgers vector and (b) the screw / edge character, as in Fig. S9a and b. (c) Cross section (depth of 20 Å) at 20 % strain showing a twin structure inside a grain; color highlights orientation to z axis. The twin begins to form at around 13 % strain and continues to grow with strain. Shaded structures represent ligament surfaces, cf. Fig. 1c.

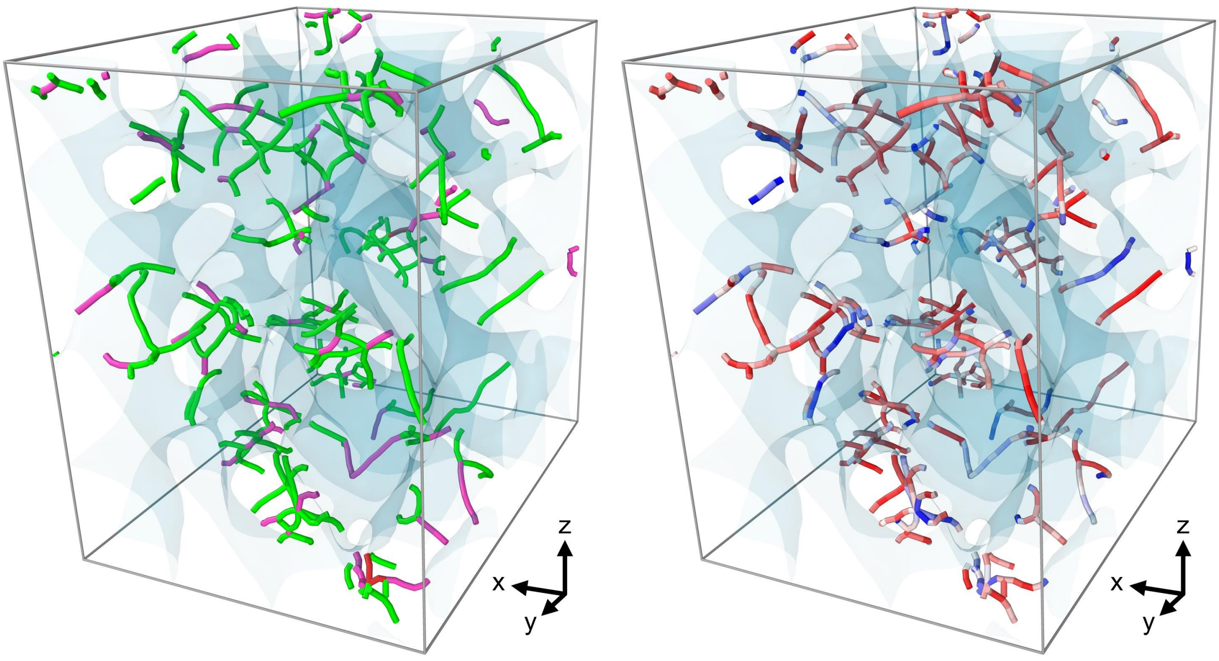

(a) (b)

FIG. S12: Snapshots showing the microstructure developing in the polycrystalline sample at 900 K. Coloring of dislocations as in Figs. S9a and b. Shaded structures represent ligament surfaces, cf. Fig. 1c.
